# Herpes Simplex Virus Type 1 and Type 2 Infection Increases Atherosclerosis Risk: Evidence Based on a Meta-Analysis

**DOI:** 10.1155/2016/2630865

**Published:** 2016-04-19

**Authors:** Yu peng Wu, Dan dan Sun, Yun Wang, Wen Liu, Jun Yang

**Affiliations:** ^1^Department of Cardiovascular Ultrasound, The First Affiliated Hospital of China Medical University, Shenyang, Liaoning 110001, China; ^2^Department of Neurosurgery, The First Affiliated Hospital of China Medical University, Shenyang, Liaoning 110001, China

## Abstract

*Objective.* The aim of our study was to evaluate the relation of herpes simplex virus type 1 (HSV-1) and type 2 (HSV-2) infection with the risk of atherosclerosis (AS).* Methods.* A systematic literature search was performed through three electronic databases. The pooled odds ratio (OR) and corresponding 95% confidence interval (CI) were used to assess the effect of HSV-1 and HSV-2 infection on AS risk.* Results.* 17 studies were available for meta-analysis of HSV-1 infection and AS risk and seven studies for meta-analysis of HSV-2 infection and AS risk. Subjects exposed to HSV-1 infection exhibited an increased risk of AS (OR = 1.77; 95% CI: 1.40–2.23; *P* < 0.001). And consistent elevated AS risks for HSV-1 positive subjects were found in all subgroup analysis of disease type, region, male proportion, and age. HSV-2 positive subjects demonstrated significantly increased AS risk (OR = 1.37; 95% CI: 1.13–1.67; *P* < 0.005). In subgroup analysis, elevated AS risks were only observed in myocardial ischemia group, male proportion >60% group, and age ≤60-year-old group.* Conclusion.* Our meta-analysis indicated that HSV-1 and HSV-2 infection could increase the risk of contracting AS.

## 1. Background

Atherosclerosis (AS) is a major public health problem worldwide that leads to various life-threatening complications, such as coronary artery disease, stroke, and peripheral artery disease [1, 2]. Traditional risk factors include hyperlipidemia, hypertension, diabetes mellitus, smoking, and a positive family history, but these do not fully explain the extent and severity of the conditions [3]. In recent years, numerous studies have implicated that pathogen burden might play an important role in the pathogenesis of atherosclerosis, for example,* Helicobacter pylori*,* Chlamydia pneumoniae*, and herpes simplex virus (HSV) [4–6].

HSV infection was widespread in the developed countries, with a prevalence of between 35% and 40% [7]. The data showed that the prevalence of HSV-1 and HSV-2 is 37% and 28%, respectively [7]. HSV was first proposed to be a risk factor for AS several decades ago when a chicken herpesvirus led to occlusive AS of large muscular arteries in an animal model [8]. Subsequent molecular biology and epidemiology studies have strengthened the hypothesis that HSV is an important risk factor in the development of AS in humans. The atherogenic mechanisms of HSV may involve increasing adherence of leukocytes to endothelium, inducing lipid accumulation in vascular smooth muscle cells (VSMCs), and contributing to deposition of thrombin in atherosclerotic plaques [9–11].

Recently an increasing number of epidemiologic studies have investigated the association between HSV infection and AS risk by testing HSV antibodies [12]. Siscovick et al. revealed that HSV-1 infection was associated with a 2-fold increase in the risk of incident MI and death from coronary heart disease [13]. Kis et al. detected increased levels of HSV-1 antibodies in patients with acute ischemic stroke, suggesting an association of HSV-1 infection with the disease [14]. The data of Guan et al. showed a higher prevalence of antibodies against HSV-2 in the subjects with acute myocardial infarction [15]. However, there were still some studies demonstrating no relationship between HSV infection and atherosclerosis [16, 17]. Given the controversial results of these studies, we deemed it necessary to conduct a quantitative and systematic assessment with rigorous methodology to further evaluate the potential role of HSV infection in the development of AS. We performed a meta-analysis to explore the relationship between HSV-1 and HSV-2 and AS risk.

## 2. Methods

### 2.1. Publication Search

We searched the databases of PubMed, Web of Science, and CNKI (China National Knowledge Infrastructure) for articles on any relationship between HSV-1 and HSV-2 infection and the risk of developing AS. The last search date was March 15, 2015. The following key terms were used: “Herpes Simplex Virus OR HSV” and “atherosclerosis OR myocardial ischemia OR ischemic heart disease OR coronary artery disease OR angina OR myocardial infarction OR stroke OR cerebral ischemia OR carotid artery disease OR peripheral artery disease.” The references cited in the research papers were further searched manually for potentially available publications.

### 2.2. Inclusion Criteria

(1) The study is a case-control design. (2) The study evaluates the association between HSV-1 and HSV-2 infection and AS risk. (3) The study confirms the diagnosis of the atherosclerotic diseases. (4) The study clearly supplies the values (or percentage) of positivity for HSV-1 and HSV-2 infection in cases and controls, respectively. (5) The study is published in English or Chinese.

### 2.3. Data Extraction

Data from these studies were extracted by two of the authors (Yu peng Wu and Dan dan Sun) independently using a standardized form, who reached a consensus on all items. The following data were collected from each study: first author, year of publication, country, region, disease type, mean age, male proportion, detection method of HSV-1 and HSV-2 infection, sample size, and the positivity or negativity for HSV-1 and HSV-2 infection in cases and controls, respectively.

### 2.4. Statistical Analysis

The pooled odds ratios (OR) with 95% confidence intervals (CI) were used to assess the association of HSV-1 and HSV-2 infection with AS risk. Statistical heterogeneity between studies was assessed with the *χ* ^2^-based *Q* test and *I* ^2^ [18]. When heterogeneity was not an issue (*P* > 0.10), a fixed-effect model with the Mantel-Haenszel method was used [19]. Otherwise, a random-effect model using the DerSimonian-Laird method was used [20]. Meanwhile, subgroup analysis was conducted for different geographic regions, male proportion, mean age, and disease types (divided into myocardial ischemia and other types of AS). To explore sources of heterogeneity across studies, we conducted logistic metaregression using the following study characteristics: region, test method, and positivity for HSV-1 and HSV-2 infection in controls. In addition, publication bias was evaluated qualitatively by performing funnel plots and was assessed quantitatively by Begg's test and Egger's test, respectively (*P* < 0.05 was considered representative of statistically significant publication bias) [21, 22]. The statistical analysis was performed using STATA 12.0 software (Stata, College Station, TX, USA).

## 3. Results

### 3.1. Study Characteristics

**Figure 1:**
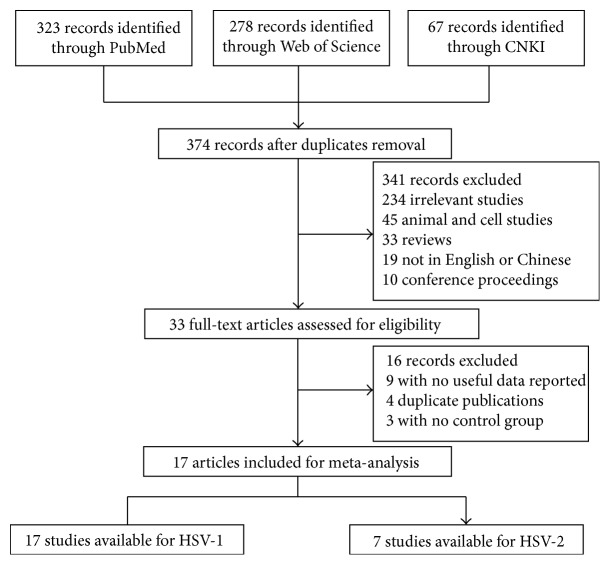
Flowchart for identification of studies.

Applying the search strategy, 374 papers were found. The titles, abstracts, and full texts of all retrieved articles were reviewed in accordance with the defined criteria. One study by Mendy et al. was excluded because the atherosclerotic disease was diagnosed by self-reported questionnaire, which could be subject to recall bias or misclassification [23]. Overall, 17 studies including a total of 3488 cases and 4241 controls were available for this analysis [6, 13–17, 24–34]. Among these, seven studies with 1810 cases and 1050 controls were available for HSV-2 infection analysis, and all 17 were available for analysis of HSV-1 infection (Figure 1).

The characteristics of the selected studies were listed in Tables 1 and 2. Of the 17 studies, 14 were published in English and three were written in Chinese. The sample sizes ranged from 15 to 1532. The controls were randomly selected and frequency-matched with the cases on age, region, and gender. Several methods were used to detect HSV-1 and HSV-2 specific antibody, including enzyme-linked immunosorbent assay (ELISA), solid-phase radioimmunoassay (SPRIA), and western blotting (WB). The diseases included myocardial ischemia, stroke, carotid artery disease, and mixed AS lesions (coronary, cerebral, carotid, and peripheral artery involvement) (Tables 1 and 2).

### 3.2. Effect of HSV-1 Infection on AS Risk

**Figure 2:**
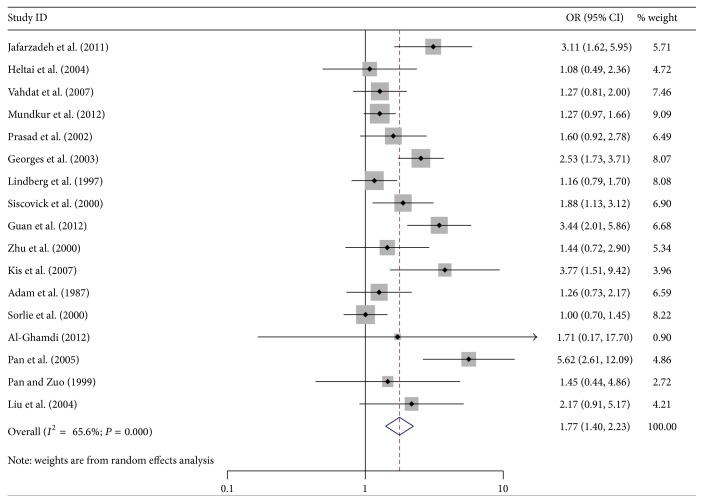
Forest plot showing the association between HSV-1 infection and atherosclerosis.

The relationships between HSV-1 and HSV-2 infection and the risk of AS were shown in Table 3. Overall, there was statistical evidence of significantly elevated AS risk associated with HSV-1 infection (OR = 1.77; 95% CI = 1.40–2.23) (Figure 2). In terms of stratified analysis by disease type, there were significant elevated risks in both myocardial ischemia and other types of AS for HSV-1 infection (myocardial ischemia: OR = 1.83; 95% CI = 1.40–2.40; other types of AS: OR = 1.73; 95% CI = 1.02–1.83). When stratified by region, AS risks were elevated in Asians, Europeans, and Americans (Asians: OR = 2.49; 95% CI = 1.57–3.97; Europeans: OR = 1.63; 95% CI = 1.09–2.42; Americans: OR = 1.32; 95% CI = 1.06–1.65). And consistent elevated AS risks for HSV-1 positive subjects were found in all subgroup analysis of age and male proportion (male proportion ≤60%: OR = 2.26; 95% CI = 1.19–4.30; male proportion >60%: OR = 1.80; 95% CI = 1.40–2.31; age ≤60-year-old: OR = 1.74; 95% CI = 1.26–2.41; age >60-year-old: OR = 2.52; 95% CI = 1.92–3.30).

### 3.3. Effect of HSV-2 Infection on AS Risk

**Figure 3:**
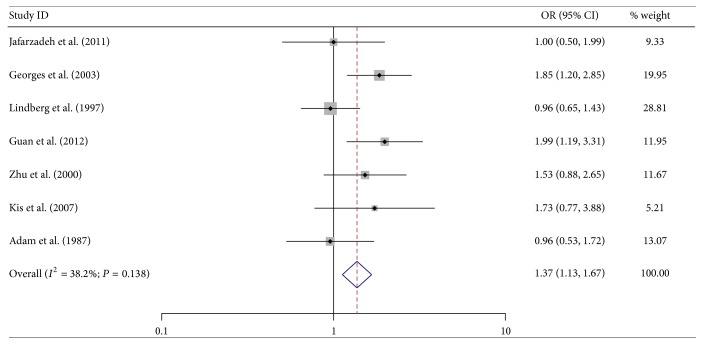
Forest plot showing the association between HSV-2 infection and atherosclerosis.

In total population, HSV-2 positive subjects demonstrated significantly elevated AS risk when compared with the negative ones (OR = 1.37; 95% CI = 1.13–1.67) (Figure 3). In terms of stratified analysis by disease type, increased risk was only observed in myocardial ischemia and not in other types of AS (myocardial ischemia: OR = 1.66; 95% CI = 1.28–2.15). We also performed stratified analysis by region, age, and male proportion. Significantly elevated risks were observed in male proportion >60% group and age ≤60-year-old group (male proportion >60%: OR = 1.61; 95% CI = 1.25–2.07; age ≤60-year-old: OR = 1.41; 95% CI = 1.08–1.85) (Table 3).

### 3.4. Heterogeneity

There was heterogeneity among studies on HSV-1 infection but not in studies on HSV-2 infection (HSV-1 infection: *P* < 0.001; *I* ^2^ = 65.60%; HSV-2 infection: *P* = 0.138; *I* ^2^ = 38.2%). To explore sources of heterogeneity across studies, we compared HSV-1 infection according to region of origin, test method, and positivity for HSV-1 infection in controls. We found that the region (*P* < 0.05), but not test method and the positivity for HSV-1 infection in controls (*P* > 0.05), might play a role in the initial heterogeneity, which could explain the *I* ^2^ value of 29% in the overall comparison of HSV-1 infection.

### 3.5. Sensitivity Analysis and Publication Bias

There was no significant difference in the pooled OR estimated by omitting one study at a time, indicating that the final results of this meta-analysis were relatively stable and reliable (see Table S1 in Supplementary Material available online at http://dx.doi.org/10.1155/2016/2630865). The Begg and Egger tests were conducted to evaluate publication bias. Both revealed no evidence of publication bias in our study; the results were shown in Table 4, Figure S1, and Figure S2.

## 4. Discussion

Herpesvirus has been implicated in the inflammatory atherosclerotic process [35]. Chronic activation of inflammation by herpesvirus infection is hypothesized to promote atherosclerosis and thrombosis. As the major subtypes of herpesvirus, HSV-1 and HSV-2 have been a concern in relation to AS for many years. However, the existing data are somewhat conflicting. Hence, we deemed it necessary to take a quantitative approach by combining the results of various studies and provide what to our knowledge is the first meta-analysis evaluating the effect of HSV-1 and HSV-2 infection on AS risk.

In the overall analysis, significant increased risk was observed for both HSV-1 and HSV-2 infection, indicating that HSV infection may play an important role in the process of atherogenesis. Some mechanistic studies may explain certain relationships. In 1991, Etingin et al. demonstrated that the endothelial cells infected by HSV might express the adhesion molecule GMP140, which could mediate endothelial cell injury and inflammation [36]. Subsequently, Chirathaworn et al. showed that HSV enhanced the uptake of oxidized low-density lipoprotein in endothelial cells [37]. The atherogenic effect of HSV not only concerned the endothelial cells but also involved VSMCs. It had been reported that more saturated cholesteryl esters and triacylglycerols accumulated in VSMCs infected by HSV than in uninfected cells [38]. In addition, Key et al. concluded that HSV could contribute to deposition of thrombi on atherosclerotic plaques and induce coagulant necrosis by decreasing thrombomodulin activity and increasing tissue factor activity [39]. These in vitro studies demonstrated that HSV exerts effects in almost every step of atherogenesis.

Subgroup analysis suggested that both HSV-1 and HSV-2 infection had a significant risk effect in myocardial ischemia. Borderline significance was found for other types of AS in HSV-1 infection whereas no association was observed between HSV-2 infection and other types of AS. Many studies have reported the detection of HSV-1 DNA in human vascular tissue from different sites. Benditt et al. first found HSV-1 DNA in human vascular tissue from the ascending aorta in patients undergoing coronary bypass surgery [40]. Subsequently, HSV-1 DNA was reported in coronary artery tissue. Chiu et al. detected HSV-1 DNA in plaques from occlusive carotid artery [41], and HSV-1 DNA was also found in atherosclerotic tissues from six types of atherosclerotic lesions by Shi and Tokunaga [42]. However, only one study, by Kotronias and Kapranos, reported the detection of HSV-2 DNA in coronary artery tissue [43]. These data might partially explain the different results from our subgroup analysis. Future studies concerning the association between HSV-2 infection and other types of AS should be performed to confirm our results.

In the stratified analysis of age and male proportion, we found no relationship of HSV-2 infection with risk of AS in male proportion ≤60% group. We presumed that gender difference might account for more than half of the reason. Males are more likely to suffer from AS than females [44]. And androgen appeared to be associated with an increased risk of coronary artery disease by adversely affecting the plasma lipid and lipoprotein profile, producing thrombosis and cardiac hypertrophy [45].

Regarding the subgroup analysis of diverse regions, HSV-1 infection had risk effects on all three subgroups of Asians, Europeans, and Americans. However, the association of HSV-2 infection with AS did not reach statistical significance in any subgroup, possibly because of the limited number of studies and relatively small sample size in each subgroup (two studies of Asians, three studies of Europeans, and two studies of Americans). More well-designed studies with larger sample sizes should be conducted for future validation.

We are aware that this meta-analysis has its own limitations. First, only seven articles with 1810 cases and 1050 controls were available for HSV-2 analysis; the relatively small number of participants made it difficult to perform stratified analysis. Second, our meta-analysis was based on unadjusted estimates; OR adjusted for age and sex should be pooled to provide exact summary estimates if more specific data from studies become available. Third, significant heterogeneity existed in the overall comparison of HSV-1 infection, although we found that regional differences may account for this heterogeneity.

## 5. Conclusions

Our meta-analysis indicated that HSV-1 and HSV-2 infection potentially increases the risk of AS. However, further large-scale and well-designed studies, including different geographic regions and careful matching between cases and controls, are required to confirm these results.

## Supplementary Material

This supplementary material consisted of one table (Table S1) and two figures (Figure S1 and Figure S2). Table S1 showed the ORs and 95% CI of sensitivity analysis concerning HSV-1 and HSV-2. It was no significant difference detected on the pooled OR which was estimated by omitting one study at a time. Figure S1 was Begg's funnel plots concerning HSV-1, and Figure S2 concerning HSV-2. The shape of the funnel plots did not reveal any evidence of obvious asymmetry in the overall meta-analysis.

## Figures and Tables

**Table 1:** Characteristics of literatures included in the meta-analysis concerning HSV-1.

First author	Year	Country	Region	Disease type	Test method	Male proportion	Age	Sample size	Case size		Control size	
									HSV-1 (+)	HSV-1 (−)	HSV-1 (+)	HSV-1 (−)
Jafarzadeh [24]	2011	Iran	Asian	Myocardial ischemia	ELISA	59%	54	180	73	47	20	40
Heltai [25]	2004	Hungary	European	Myocardial ischemia	ELISA	—	—	129	59	24	32	14
Vahdat [26]	2007	Iran	Asian	Myocardial ischemia	ELISA	49%	—	1754	198	24	1327	205
Mundkur [17]	2012	UK	European	Myocardial ischemia	ELISA	68%	53	866	240	193	214	219
Prasad [27]	2002	America	American	Myocardial ischemia	ELISA	61%	56	375	211	35	102	27
Georges [28]	2003	Germany	European	Myocardial ischemia	ELISA	73%	62	1324	922	69	280	53
Lindberg [29]	1997	Sweden	European	Carotid artery disease	SPRIA	—	—	534	198	69	190	77
Siscovick [13]	2000	America	American	Myocardial ischemia	WB	63%	74	618	191	22	333	72
Guan [15]	2012	China	Asian	Myocardial ischemia	ELISA	62%	56	252	71	31	60	90
Zhu [30]	2000	America	American	Myocardial ischemia	ELISA	63%	57	233	133	25	59	16
Kis [14]	2007	Hungary	European	Stroke	ELISA	58%	52	111	24	35	8	44
Adam [31]	1987	America	American	Mixed AS lesions	SPRIA	100%	58	226	75	38	69	44
Sorlie [16]	2000	America	American	Myocardial ischemia	SPRIA	52%	55	726	166	55	379	126
Al-Ghamdi [6]	2012	Saudi Arabia	Asian	Mixed AS lesions	ELISA	64%	56	90	72	3	14	1
Pan [32]	2005	China	Asian	Myocardial ischemia	ELISA	48%	62	127	46	16	22	43
Pan [33]	1999	China	Asian	Myocardial ischemia	ELISA	70%	—	60	24	6	22	8
Liu [34]	2004	China	Asian	Myocardial ischemia	ELISA	73%	67	124	72	21	19	12

HSV-1: herpes simplex virus type 1; ELISA: enzyme-linked immunosorbent assay; SPRIA: solid-phase radioimmunoassay; WB: western blot; AS: atherosclerosis.

**Table 2:** Characteristics of literatures included in the meta-analysis concerning HSV-2.

First author	Year	Country	Region	Disease type	Test method	Male proportion	Age	Sample size	Case size		Control size	
									HSV-2 (+)	HSV-2 (−)	HSV-2 (+)	HSV-2 (−)
Jafarzadeh [24]	2011	Iran	Asian	Myocardial ischemia	ELISA	59%	54	180	34	86	17	43
Georges [28]	2003	Germany	European	Myocardial ischemia	ELISA	73%	62	1324	139	852	27	306
Lindberg [29]	1997	Sweden	European	Carotid artery disease	SPRIA	—	—	534	64	203	66	201
Guan [15]	2012	China	Asian	Myocardial ischemia	ELISA	62%	56	252	56	46	57	93
Zhu [30]	2000	America	American	Myocardial ischemia	ELISA	63%	57	233	84	74	32	43
Kis [14]	2007	Hungary	European	Stroke	ELISA	58%	52	111	23	36	14	38
Adam [31]	1987	America	American	Mixed AS lesions	SPRIA	100%	58	226	30	83	31	82

HSV-2: herpes simplex virus type 2; ELISA: enzyme-linked immunosorbent assay; SPRIA: solid-phase radioimmunoassay; AS: atherosclerosis.

**Table 3:** Pooled OR and 95% CI of stratified meta-analysis.

Variables	HSV-1					HSV-2				
	*N*	OR (95% CI)	*P* value	*I* ^2^ (%)	*P*_Het_	*N*	OR (95% CI)	*P* value	*I* ^2^ (%)	*P*_Het_
Total	17	**1.77 (1.40–2.23)**	**<0.001**	65.6	<0.001	7	**1.37 (1.13–1.67)**	**0.002 **	38.2	0.138
Disease type										
Myocardial ischemia	13	**1.83 (1.40–2.40)**	**<0.001**	69.6	<0.001	4	**1.66 (1.28–2.15)**	**<0.001**	<0.001	0.409
Other types of AS	4	**1.37 (1.02–1.83)**	**0.035**	45.8	0.137	3	1.05 (0.77–1.41)	0.777	<0.001	0.410
Region										
Asian	7	**2.49 (1.57–3.97)**	**<0.001 **	61.3	0.017	2	1.47 (0.75–2.86)	0.261	59.4	0.116
European	5	**1.63 (1.09–2.42)**	**0.017 **	73.2	0.005	3	1.40 (0.87–2.26)	0.168	61.9	0.072
American	5	**1.32 (1.06–1.65)**	**0.014 **	11.9	0.338	2	1.22 (0.82–1.83)	0.323	22.2	0.257
Male proportion										
≤60%	5	**2.26 (1.19–4.30)**	**0.013 **	84.0	<0.001	2	1.26 (0.75–2.13)	0.381	3.6	0.308
>60%	10	**1.80 (1.40–2.31)**	**<0.001**	47.9	0.044	4	**1.61 (1.25–2.07)**	**<0.001**	26.8	0.251
Age										
≤60-year-old	9	**1.74 (1.26–2.41)**	**0.001 **	67.4	0.002	5	**1.41 (1.08–1.85)**	**0.012 **	14.9	0.320
>60-year-old	4	**2.52 (1.92–3.30)**	**<0.001**	46.5	0.132	1	1.85 (1.20–2.85)	0.005	—	—

HSV-1: herpes simplex virus type 1; HSV-2: herpes simplex virus type 2; AS: atherosclerosis; OR: odds ratio; *P*_Het_: the *P* value of heterogeneity; —: no data. The results were in bold, if the 95% CI excluded 1 or *P* < 0.05.

**Table 4:** The results of Begg's and Egger's test for publication bias.

HSV type	Begg's test		Egger's test	
	*Z* value	*P* value	*t* value	*P* value
HSV-1	1.69	0.091	1.71	0.107
HSV-2	0.00	1.000	0.15	0.887

HSV: herpes simplex virus; HSV-1: herpes simplex virus type 1; HSV-2: herpes simplex virus type 2.
