# Supplementary material for: Herpes Simplex Virus Type 1 and Type 2 Infection Increases Atherosclerosis Risk: Evidence Based on a Meta-Analysis
Source: Biomed Res Int. 2016 Apr 19;2016:2630865. doi: 10.1155/2016/2630865 (PMC4853930; doi:10.1155/2016/2630865)
Supplement: Supplementary file 1 — This supplementary material consisted of one table (Table S1) and two figures (Figure S1 and Figure S2). Table S1 showed the ORs and 95% CI of sensitivity analysis concerning HSV-1 and HSV-2. It was no significant difference detected on the pooled OR which was estimated by omitting one study at a time. Figure S1 was Begg's funnel plots concerning HSV-1, and Figure S2 concerning HSV-2. The shape of the funnel plots did not reveal any evidence of obvious asymmetry in the overall meta-analysis. [file 2630865.f1.doc]

**Supplement data**

**Figure S1.** **Begg's funnel plot for publication bias test of HSV-1 infection.**

**
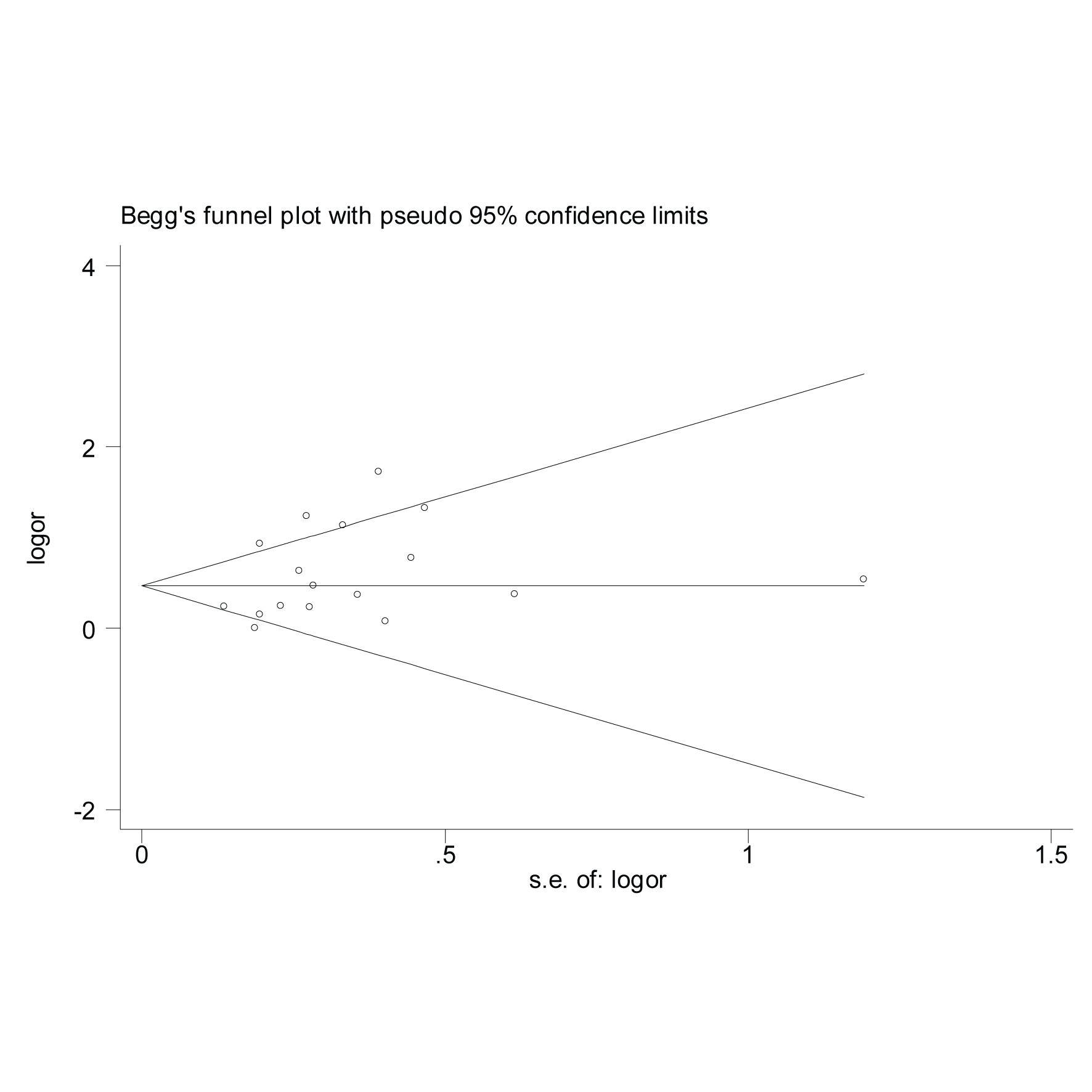
**

Each point represents a separate study for the indicated association. s.e: standardized effect.

**Figure S2. Begg's funnel plot for publication bias test of HSV-2 infection.**

**
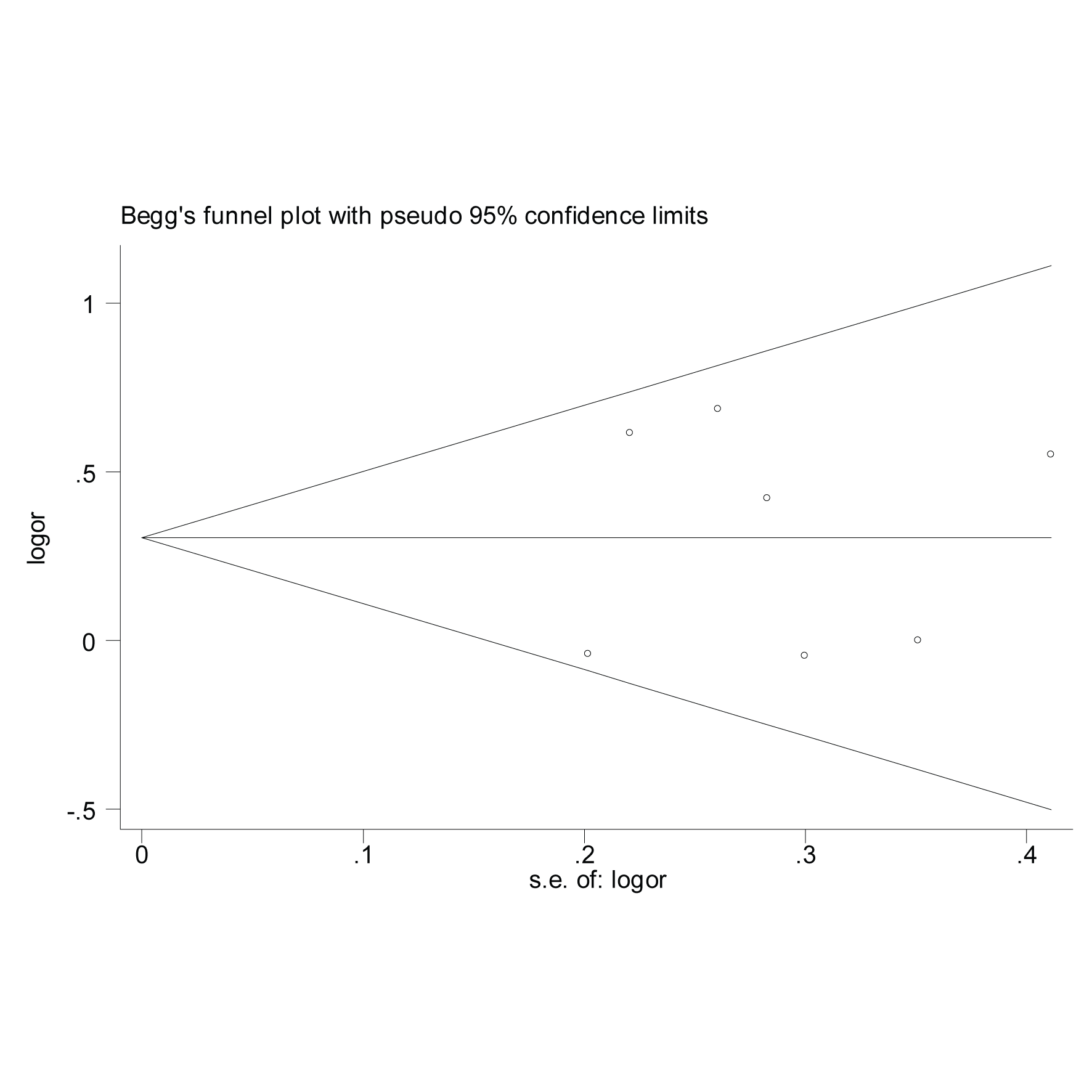
**

Each point represents a separate study for the indicated association. s.e: standardized effect.

**Table S1. ORs (95% CI) of sensitivity analysis.**

| Excluding literature | HSV-1 | HSV-2 |
| --- | --- | --- |
| one by one | OR (95% CI) | OR (95% CI) |
| Over all | 1.77(1.40-2.23) | 1.37(1.13-1.67) |
| Jafarzadeh, A. (2011) | 1.71(1.35-2.16) | 1.41(1.15-1.73) |
| Heltai, K. (2004) | 1.82(1.43-2.31) |  |
| Vahdat, K. (2007) | 1.82(1.42-2.33) |  |
| Mundkur, L. A. (2012) | 1.83(1.42-2.37) |  |
| Prasad, A. (2002) | 1.78(1.40-2.29) |  |
| Georges, J. L. (2003) | 1.71(1.35-2.17) | 1.25(1.00-1.56) |
| Lindberg, G. (1997) | 1.83(1.44-2.35) | 1.54(1.22-1.93) |
| Siscovick, D. S. (2000) | 1.77(1.38-2.26) |  |
| Guan, X. (2012) | 1.67(1.34-2.10) | 1.29(1.04-1.59) |
| Zhu, J. (2000) | 1.79(1.41-2.29) | 1.35(1.09-1.66) |
| Kis, Z. (2007) | 1.71(1.36-2.16) | 1.35(1.10-1.65) |
| Adam, E. (1987) | 1.82(1.42-2.32) | 1.43(1.16-1.76) |
| Sorlie, P.D. (2000) | 1.86(1.47-2.35) |  |
| Al-Ghamdi, A. (2012) | 1.77(1.40-2.24) |  |
| Pan, S.Z. (2005) | 1.65(1.34-2.05) |  |
| Pan, J.H. (1999) | 1.78(1.40-2.26) |  |
| Liu, D.N. (2004) | 1.76(1.38-2.23) |  |

**Checklist S1. PRISMA checklist.**

| **Section/topic** | **#** | **Checklist item** | **Reported on page #** |
| --- | --- | --- | --- |
| **TITLE** | | |  |
| Title | 1 | Potential diagnostic value of serum p53 antibody for detecting esophageal cancer: a meta-analysis | Page1 **Herpes simplex virus type 1 and type 2 infection increases atherosclerosis risk: evidence based on a meta-analysis** |
| **ABSTRACT** | | |  |
| Structured summary | 2 | Provide a structured summary including, as applicable: background; objectives; data sources; study eligibility criteria, participants, and interventions; study appraisal and synthesis methods; results; limitations; conclusions and implications of key findings; systematic review registration number. | Page 2-3 **Abstract** |
| **INTRODUCTION** | | |  |
| Rationale | 3 | Describe the rationale for the review in the context of what is already known. | Page 3-4 **Introduction** |
| Objectives | 4 | Provide an explicit statement of questions being addressed with reference to participants, interventions, comparisons, outcomes, and study design (PICOS). | Page 4 **Introduction** |
| **METHODS** | | |  |
| Protocol and registration | 5 | Indicate if a review protocol exists, if and where it can be accessed (e.g., Web address), and, if available, provide registration information including registration number. | NA |
| Eligibility criteria | 6 | Specify study characteristics (e.g., PICOS, length of follow-up) and report characteristics (e.g., years considered, language, publication status) used as criteria for eligibility, giving rationale. | Page 4 **Methods: Publication search** |
| Information sources | 7 | Describe all information sources (e.g., databases with dates of coverage, contact with study authors to identify additional studies) in the search and date last searched. | Page 4 **Methods: Publication search** |
| Search | 8 | Present full electronic search strategy for at least one database, including any limits used, such that it could be repeated. | Page 4 **Methods: Publication search** |
| Study selection | 9 | State the process for selecting studies (i.e., screening, eligibility, included in systematic review, and, if applicable, included in the meta-analysis). | Page 4-5 **Methods: Inclusion criteria** |
| Data collection process | 10 | Describe method of data extraction from reports (e.g., piloted forms, independently, in duplicate) and any processes for obtaining and confirming data from investigators. | Page 5 **Methods: Data extraction** |
| Data items | 11 | List and define all variables for which data were sought (e.g., PICOS, funding sources) and any assumptions and simplifications made. | Page 5 **Methods: Data extraction** |
| Risk of bias in individual studies | 12 | Describe methods used for assessing risk of bias of individual studies (including specification of whether this was done at the study or outcome level), and how this information is to be used in any data synthesis. | Page 5 **Methods: Statistical analysis** |
| Summary measures | 13 | State the principal summary measures (e.g., risk ratio, difference in means). | Page 5 **Methods: Statistical analysis** |
| Synthesis of results | 14 | Describe the methods of handling data and combining results of studies, if done, including measures of consistency (e.g., I^2^) for each meta-analysis. | Page 5 **Methods: Statistical analysis** |

Page 1 of 2

| **Section/topic** | **#** | **Checklist item** | **Reported on page #** |
| --- | --- | --- | --- |
| Risk of bias across studies | 15 | Specify any assessment of risk of bias that may affect the cumulative evidence (e.g., publication bias, selective reporting within studies). | Page 6 **Methods: Statistical analysis** |
| Additional analyses | 16 | Describe methods of additional analyses (e.g., sensitivity or subgroup analyses, meta-regression), if done, indicating which were pre-specified. | Page 6 **Methods: Statistical analysis** |
| **RESULTS** | | |  |
| Study selection | 17 | Give numbers of studies screened, assessed for eligibility, and included in the review, with reasons for exclusions at each stage, ideally with a flow diagram. | Page 6 **Results: Study characteristics; Fig. 1** |
| Study characteristics | 18 | For each study, present characteristics for which data were extracted (e.g., study size, PICOS, follow-up period) and provide the citations. | Page 6-7 **Results: Study characteristics; Table 1** |
| Risk of bias within studies | 19 | Present data on risk of bias of each study and, if available, any outcome level assessment (see item 12). | Page 6-7 **Results: Study characteristics; Table 1** |
| Results of individual studies | 20 | For all outcomes considered (benefits or harms), present, for each study: (a) simple summary data for each intervention group (b) effect estimates and confidence intervals, ideally with a forest plot. | Page 6-7 **Results: Study characteristics; Table 1** |
| Synthesis of results | 21 | Present results of each meta-analysis done, including confidence intervals and measures of consistency. | Page 7 **Results:** Effect of HSV-1 and HSV-2 infection on AS risk**; Table 2, Figure 2-3** |
| Risk of bias across studies | 22 | Present results of any assessment of risk of bias across studies (see Item 15). | Page 8 **Results:** Sensitivity analysis and publication bias**; Table 3, Figure S1-S2** |
| Additional analysis | 23 | Give results of additional analyses, if done (e.g., sensitivity or subgroup analyses, meta-regression [see Item 16]). | Page 8 **Meta-regression, Results** Heterogeneity**; Subgroup analyses, Table 2; Sensitivity, Table S1.** |
| **DISCUSSION** | | |  |
| Summary of evidence | 24 | Summarize the main findings including the strength of evidence for each main outcome; consider their relevance to key groups (e.g., healthcare providers, users, and policy makers). | Page 8-11 **Discussion** |
| Limitations | 25 | Discuss limitations at study and outcome level (e.g., risk of bias), and at review-level (e.g., incomplete retrieval of identified research, reporting bias). | Page 10-11 **Discussion** |
| Conclusions | 26 | Provide a general interpretation of the results in the context of other evidence, and implications for future research. | Page 11 **Conclusion** |
| **FUNDING** | | |  |
| Funding | 27 | Describe sources of funding for the systematic review and other support (e.g., supply of data); role of funders for the systematic review. | NA |
